# Response to DNA damage: why do we need to focus on protein phosphatases?

**DOI:** 10.3389/fonc.2013.00008

**Published:** 2013-01-31

**Authors:** Midori Shimada, Makoto Nakanishi

**Affiliations:** Department of Cell Biology, Graduate School of Medical Sciences, Nagoya City UniversityNagoya, Japan

**Keywords:** DNA damage response, phosphorylation, protein phosphatase, DNA repair, chromatin

## Abstract

Eukaryotic cells are continuously threatened by unavoidable errors during normal DNA replication or various sources of genotoxic stresses that cause DNA damage or stalled replication. To maintain genomic integrity, cells have developed a coordinated signaling network, known as the DNA damage response (DDR). Following DNA damage, sensor molecules detect the presence of DNA damage and transmit signals to downstream transducer molecules. This in turn conveys the signals to numerous effectors, which initiate a large number of specific biological responses, including transient cell cycle arrest mediated by checkpoints, DNA repair, and apoptosis. It is recently becoming clear that dephosphorylation events are involved in keeping DDR factors inactive during normal cell growth. Moreover, dephosphorylation is required to shut off checkpoint arrest following DNA damage and has been implicated in the activation of the DDR. Spatial and temporal regulation of phosphorylation events is essential for the DDR, and fine-tuning of phosphorylation is partly mediated by protein phosphatases. While the role of kinases in the DDR has been well documented, the complex roles of protein dephosphorylation have only recently begun to be investigated. Therefore, it is important to focus on the role of phosphatases and to determine how their activity is regulated upon DNA damage. In this work, we summarize current knowledge on the involvement of serine/threonine phosphatases, especially the protein phosphatase 1, protein phosphatase 2A, and protein phosphatase Mg^2+^/Mn^2+^-dependent families, in the DDR.

## INTRODUCTION

The DNA damage response (DDR) signaling network mediates a wide variety of cellular events, including DNA repair, cell cycle arrest, apoptosis, and premature senescence, to maintain genomic integrity. Loss of checkpoint function results in chromosomal instability and aneuploidy, which promote tumorigenesis, suggesting that proper checkpoint signaling is essential for preventing cancer. From recent studies, it has become clear that protein phosphorylation plays a major role in the regulation of diverse DDR pathways. The initiation of some DDR processes is mainly mediated by protein kinases in the phosphoinositide 3-kinase (PI3-K)-related kinase family, as well as ataxia-telangiectasia mutated (ATM), ATM and Rad3-related (ATR), and DNA-dependent protein kinase (DNA-PK). These kinases orchestrate the cellular responses to DNA damage and activate the multiple cascades involved in the DDR through phosphorylation of a variety of substrates (Shiloh, 2003). Effector kinases, Chk1 and Chk2, are activated mainly by ATR and ATM, respectively, and transmit signals to a variety of downstream factors, such as p53, pRB, and Cdc25, ultimately leading to inactivation of cyclin-dependent kinases (Cdks) and inhibiting cell-cycle progression. Thus, studies have demonstrated that protein phosphorylation, mainly at serine (S)/threonine (T) residues, is essential for the DDR and regulates enzymatic activity, localization, protein–protein interactions, and stabilization. Given the fact that ATM and ATR are known to have hundreds of substrates, a large number of phosphorylation events are regulated and have roles in the DDR, although the biological significance of many of these phosphorylation events is largely unknown. Indeed, the catalytic subunits of protein phosphatases and their regulators are also targets of ATM and ATR, suggesting that the activity of protein phosphatases is regulated by phosphorylation during the DDR.

Recent large proteomic analyses have revealed that most phosphorylation events are tightly regulated both spatially and temporally, suggesting that a comprehensive analysis of the timing and location of phosphorylation events, rather than analysis of phosphorylation levels in whole cells, is essential to understand the DDR. In recent years, it has become increasingly clear that phosphatases, particularly S/T phosphatases, regulate the DDR not only by counteracting the function of kinases, but also by initiation of specific steps during the DDR. Given that fine-tuning of phosphorylation events is partly mediated by phosphatases, studies should focus on the involvement of protein phosphatases in the DDR and how their activity is regulated *in vivo*. In addition to phosphorylation, different types of post-translational modifications also have important roles in the DDR. Thus, it will be important to investigate the correlation between phosphorylation/dephosphorylation and other types of modifications. In this study, we summarize recent reports that have revealed new functions of S/T phosphatases in the DDR.

## INTRODUCTION TO THE DDR

Upon formation of DNA double-strand breaks (DSBs), ATM is autophosphorylated at S1981 and then dissociates from an inactive homodimer into active monomers (Bakkenist and Kastan, 2003). It has been reported that phosphorylation of ATM on S367, S1893, and S1981 is required for ATM activation in human cells (Bakkenist and Kastan, 2003; Kozlov et al., 2006). Contrary to these findings, corresponding phosphorylation of these sites are dispensable for murine ATM activation (Pellegrini et al., 2006; Daniel et al., 2008). Instead of ATM activation, S1981 phosphorylation is required for ATM retention at sites of DSBs through interaction with mediator of DNA damage checkpoint 1 (MDC1; So et al., 2009). ATM is recruited to DSBs and activated by the MRN complex (Mre11, Rad50, and NBS1) through the association with NBS1 (Uziel et al., 2003; Falck et al., 2005; Lee and Paull, 2005). In response to DSBs, ATM phosphorylates histone H2AX at S139 (termed γ-H2AX), which extends up to megabases away from break sites that are readily visible as foci by immunofluorescence microscopy (Burma et al., 2001; Ward and Chen, 2001; Shroff et al., 2004; Stiff et al., 2004). These γ-H2AX foci are colocalized with many proteins involved in the DDR, such as MDC1, 53BP1, BRCA1, and the MRN complex (Paull et al., 2000). Indeed, γ-H2AX is required for the recruitment and maintenance of these factors at damaged sites in order to transmit signals to downstream factors.

Following single-stranded DNA (ssDNA) damage, replication defects, or during the process of DSBs, ATR, which forms a complex with ATRIP, is recruited to the region of replication protein A (RPA)-coated ssDNA (Zou and Elledge, 2003). Rad17, associated with small subunits of replication factor C (RFC), recognizes the junctions between ssDNA and dsDNA and facilitates the loading of the Rad9-Rad1-Hus1 (9-1-1 complex) sliding clamp onto the DNA (Kondo et al., 2001; Melo et al., 2001; Zou and Elledge, 2003). In concert with ATR/ATRIP and RPA, the 9-1-1 complex appears to act as a sensor of DNA damage and interacts with TopBP1, thereby loading it onto sites of DNA damage. Rad17 interacts with Claspin to promote the phosphorylation of Chk1 (Roos-Mattjus et al., 2002; Bermudez et al., 2003; Wang et al., 2006). All of these proteins, i.e., ATRIP, RPA, RAD17, the 9-1-1 complex, and TopBP1, are phosphorylated by ATR during checkpoint activation. Through an interaction with TopBP1, ATR becomes fully active, thereby inducing subsequent responses (Delacroix et al., 2007; Lee et al., 2007a).

ATR and ATM phosphorylate and activate the effector kinases Chk1 and Chk2, respectively. Chk1 is composed of N-terminal catalytic domain and a regulatory C-terminus, which negatively regulates Chk1 kinase activity. Several reports have identified the functional roles of Chk1 phosphorylation *in vivo*. In response to cellular stresses, Chk1 phosphorylation occurs primarily on 2 residues, S317 and S345 (Liu et al., 2000; Zhao and Piwnica-Worms, 2001). Phosphorylated Chk1 is released from chromatin and accumulates in the cytoplasm to prevent activation of Cdk1 and entry into mitosis (Kramer et al., 2004). Phosphorylation of the C-terminal residues (mainly S317 and S345) block intramolecular interactions, reversing this auto-inhibition mechanism (Katsuragi and Sagata, 2004). Regardless of this negative regulatory mechanism, Chk1 has basal activity in its unmodified form, and this activity is sufficient to phosphorylate several substrates, including histone H3 at T11 and Aurora B (Falck et al., 2005; Shimada et al., 2008). Thus, phosphorylation of S317 and S345 induces conformational changes that permit full activation and spatiotemporal regulation of Chk1 (Katsuragi and Sagata, 2004; Ng et al., 2004; Clarke and Clarke, 2005; Smits et al., 2006; Loffler et al., 2007; Niida et al., 2007). Upon DSBs, Chk2 is phosphorylated at T68 by ATM, which triggers Chk2 dimerization and activation by autophosphorylation of residues T383 and T387 in the T-loop (Ahn et al., 2000; Melchionna et al., 2000; Xu et al., 2002). Given that chromatin-bound Chk2 is dissociated from chromatin in response to ionizing radiation (IR), this notion also suggests that Chk2, when localized other than undamaged sites, may allow further activation of downstream effectors (Li and Stern, 2005). Although biochemical analyses revealed that Chk2 can phosphorylate Cdc25A, Cdc25C, BRCA1, and p53, examination of Chk2-deficient mice and cells showed that Chk2 functions mainly in p53-dependent apoptosis (Jack et al., 2002). It is noteworthy that Chk2 and Chk1 have partially redundant roles and share multiple substrates (Bartek and Lukas, 2003; Uziel et al., 2003; Lee and Paull, 2005). However, despite their overlapping roles in checkpoint signaling, the biological requirements for Chk1 and Chk2 function are strikingly different (Bartek and Lukas, 2003). In any case, these checkpoint kinases phosphorylate effector molecules, such as p53 and Cdc25 proteins, to induce cell cycle arrest (Sanchez et al., 1997; Hirao et al., 2000; Shieh et al., 2000; Falck et al., 2001).

DNA-dependent protein kinase plays a critical role in DNA damage repair, especially in non-homologous end-joining (NHEJ) repair of DSBs. DNA-PK is composed of three factors, a catalytic subunit (DNA-PKcs), Ku70, and Ku80, the latter two of which form a Ku heterodimer, and is essential for NHEJ to repair DNA DSBs. The Ku70/Ku80 heterodimer first binds to each broken DNA strand, after which DNA-PKcs is recruited to the DNA ends through interaction with the Ku heterodimer (Gottlieb and Jackson, 1993), promoting DNA repair. Importantly, DNA-PKcs is autophosphorylated at multiple sites and the regions of 2023–2056 and 2609–2647 are identified as major autophosphorylation clusters (Ding et al., 2003; Block et al., 2004; Chen et al., 2005; Cui et al., 2005). Among them, S2056 and T2609 are phosphorylated following IR and extensively studied. Such autophosphorylation of DNA-PKcs is important for end processing, disassembly, and inactivation (Merkle et al., 2002). Functional analysis using hypo- or hyperphosphorylated mutations of DNA-PKcs suggest that timely phosphorylation and dephosphorylation are essential for its function (Chan et al., 2002).

## DYNAMIC CHANGES IN PROTEIN PHOSPHORYLATION FOLLOWING DNA DAMAGE

Protein phosphorylation is one of the most common post-translational modifications and is known to control many cellular processes. The phosphorylation state of a protein represents a balance between the activity of protein kinases and protein phosphatases. It has been reported that one-third of cellular proteins are phosphorylated, and more than 98% of protein phosphorylation occurs on S and T residues (Olsen et al., 2006). Recently, several large-scale proteomic studies have revealed that ATM and ATR phosphorylate hundreds of proteins, which are involved in proliferation, cell structure, transcription, metabolic signaling, and RNA splicing (Matsuoka et al., 2007; Smolka et al., 2007; Stokes et al., 2007; Virshup and Shenolikar, 2009). Thus, ATM and ATR coordinate a much wider variety of cellular activities than initially expected. Protein phosphatase catalytic subunits and a number of their regulators were identified by these screens, suggesting that they play a role in the DDR downstream of ATM/ATR, although the functional meaning of these phosphorylation events has not been investigated. Large phosphoproteomic analyses performed in later studies reported dynamic and temporal aspects of phosphorylation and dephosphorylation following DSBs (Bennetzen et al., 2010; Bensimon et al., 2010). Bennetzen et al. (2010) classified the temporal profiles of nearly 600 regulated phosphorylation sites on 209 proteins and revealed that sites phosphorylated shortly after DSBs are enriched in SQ motifs, which are targets of ATM/ATR/DNA-PK, and in novel SXXQ motifs. Importantly, they identified a considerable number of sites that are dephosphorylated immediately after DNA damage. Bensimon et al. (2010) also performed quantitative phosphoproteomics and showed that 40% of DSB-induced phosphorylation events are ATM-independent. In addition, among ATM-dependent phosphorylation events, 75% are not located in SQ/TQ motifs, indicating the involvement of additional kinases activated by ATM. Similar to the results described by Bennetzen et al. (2010), Bensimon et al. (2010) found that more than 300 sites are dephosphorylated following DSBs among approximately 750 regulated phosphorylation sites on nearly 400 proteins; however, the functions of most of these dephosphorylation events have not yet been identified. Protein phosphatases contribute to shutting off DSB-induced phosphorylation during the late DDR; thus, these proteomic analyses suggested an additional function of phosphatases, which play a primary role in initiating some DDR processes.

## PROTEIN PHOSPHATASES

The mammalian genome encodes nearly 500 protein kinases, 400 of which are S/T kinases. In contrast, the number of protein phosphatase catalytic subunits (e.g., catalytic subunit of PP1 is referred as PP1C) has been estimated to be 147, of which only about 40 are S/T phosphatases (Moorhead et al., 2007). The fact that so few S/T phosphatases counteract hundreds of distinct S/T kinases can be explained by the ability of phosphatases to form distinct components *in vivo*. Based on sequence, structure, and biological properties, S/T phosphatases can be classified into Mg^2+^/Mn^2+^-dependent phosphatases (PPMs) and the more diverse phosphoprotein phosphatases (PPPs). Among the PPP family, PP1 and PP2A are the most abundant isoforms, and their substrates have been relatively well characterized. PP1 and PP2A catalytic subunits interact with a vast number of regulators that target them to specific locations, mediate substrate specificity, and fine-tune phosphatase activity. In fact, mammalian cells contain more than 600 distinct PP1 complexes and approximately 70 PP2A holoenzymes (Ding et al., 2003).

### PP1

PP1 catalyzes the majority of protein dephosphorylation events that regulate diverse cellular processes, such as neuronal signaling, muscle contraction, glycogen synthesis, and cell proliferation. Mammals have three PP1 catalytic genes, PP1α, -γ, and -δ, which encode very closely related proteins showing more than 85% similarity, with minor differences primarily at their NH_2_ and COOH termini (Cohen, 2002). PP1γ has 2 isoforms, γ1 and γ2, generated by differential splicing of PP1γ. PP1 isoforms are expressed in all tissues and are widely distributed, except for PP1γ2, which is found only in the testes (Shima et al., 1993). PP1 isoforms show distinct subcellular localization, suggesting distinct roles and substrates for these enzymes (Andreassen et al., 1998). However, only a few reports have demonstrated specific differences for PP1 isoforms, since they do not show strict substrate specificities *in vitro* and have overlapping functions in most cases. Specificity is provided to PP1C through association with a large number of regulatory subunits that target catalytic subunits to specific subcellular localization, modulate their activity, and determine substrate specificity. Importantly many proteins involved in the DDR, including BRCA1, pRB, 53BP1, and Cdc25, harbor a PP1c-binding motif, RVxF (Ding et al., 2003; Kuntziger et al., 2011), and are targets of PP1.

The activity of PP1 is regulated by regulatory subunits such as protein phosphatase 1 nuclear targeting subunit (PNUTS), nuclear inhibitor of protein phosphatase 1 (NIPP1), inhibitor 2 (I2), and recruits PP1 onto mitotic chromatin at anaphase (Repo-Man). Most forms of regulation are also achieved through the regulatory subunits; however, phosphorylation of PP1 by Cdk is also important for its activity. Nuclear PP1 shows higher activity in G_0_/G_1_ and G_2_/M, and this change can be explained by Cdk-dependent phosphorylation of PP1 in its C-terminus. Cdk phosphorylates PP1α on T320, reducing its activity (Dohadwala et al., 1994; Berndt et al., 1997; Kwon et al., 1997). The equivalent T residue is conserved in all three PP1 isoforms (T316 in PP1β and T311 in PP1γ), and indeed, PP1γ is also inactivated by Cdk-dependent T311 phosphorylation (Shimada et al., 2010). Mice with depleted PP1γ are viable, but males show defective spermiogenesis and are infertile (Varmuza et al., 1999). These findings suggest that PP1α and/or -β could compensate for the depletion of PP1γ in development, but not in the specific function of spermiogenesis.

### THE PP2A FAMILY

PP2 is further divided into three groups on the basis of metal-dependence: metal-independent PP2A, PP4, PP5, and PP6; Ca^2+^-dependent PP2B and PP7; and Mg^2+^/Mn^2+^-dependent PP2C. Among the metal-independent group members, PP4 and PP6 share high homology with PP2A and are referred as PP2A-like phosphatases. Recent reports revealed that PP2A-like phosphatases have overlapping substrates and roles in the DDR.

#### PP2A

PP2A often functions as a heterotrimer, comprising three subunits designated A, B, and C. The core enzyme consists of the catalytic C subunit, scaffold A subunit, and variable regulatory B subunit. The regulatory B subunit defines the substrate specificity of the PP2A holoenzyme and comprises four families: PR55/B (B55), PR61/ B′ (B56), PR72/B′′, and striatins/SG2NA/B′′′. Each one of these families contains various isoforms, which, when combined with the isoforms of both A and C subunits, produce a variety of PP2A holoenzymes that perform distinct functions. Depletion or inhibition of PP2A activity in *Xenopus* egg extracts inhibits the initiation of DNA replication by preventing binding of the initiation factor Cdc45 onto prereplication complexes (Lin et al., 1998; Chou et al., 2002). Additionally, recent studies have uncovered important roles for PP2A in the DDR. For example, PP2A is essential for the activation of ATM, ATR, Chk1, Chk2, and p53 and mediates G_2_/M checkpoint control through regulation of the phosphorylation states of these proteins, although phosphorylation of Chk2 at T68, ATR at S428, and Chk1 at S317 is observed even in irradiated cells lacking PP2A activity (Yan et al., 2010).

#### PP4

PP4 is structurally and functionally related to PP2A and shares 65% amino acid identity with PP2A. PP4c associates with the regulatory subunits PP4R1, PP4R2, PP4R3α, PP4R3β, and PP4R4 (Cohen et al., 2005; Chen et al., 2008). Recent reports have demonstrated that PP4 possesses various cellular functions, including roles in nucleation, growth, and stabilization of microtubules at centrosomes/spindle bodies during cell division (Brewis et al., 1993; Helps et al., 1998; Hastie et al., 2000; Sumiyoshi et al., 2002). PP4 is localized predominantly to the nucleus, but some PP4 is present in cytoplasm and in mitotic centrosomes. Importantly, depletion of PP4 leads to embryonic lethality in mice, and PP4 deficiency in thymocytes results in decreased proliferation, indicating the essential role of PP4 in development and cell growth (Shui et al., 2007). Thanks to the functional analysis of newly identified PP4 substrates, our understanding of the role of PP4 in the DDR has also grown. Indeed, PP4 is involved in recovery from the G2/M checkpoint arrest after IR and required for cell survival in the presence of DNA replication inhibitors (Chowdhury et al., 2008; Nakada et al., 2008).

#### PP5

Unlike other related phosphatases, PP5 contains tetratricopeptide repeat (TPR) domains at the N-terminus. A catalytic domain and an auto-inhibitory domain are located at the C-terminus (Chinkers, 2001). Moreover, unlike other related phosphatases, whose substrate specificity is mediated by regulatory subunits, PP5 is regulated by protein–protein interactions through the TPR motif. Thus far, PP5 has been reported to interact with several proteins involved in the regulation of steroid signaling (Chen et al., 1996; Silverstein et al., 1997), cell cycle progression (Ollendorff and Donoghue, 1997; Zuo et al., 1998), and apoptosis (Morita et al., 2001) via the TPR domain. In addition, PP5 has been reported to be less abundant than other phosphatases, and has been shown to have low basal activity (Chinkers, 2001). PP5 knockout mice are viable, and the replication checkpoint is intact; however, the G_2_/M checkpoint is impaired in PP5-knockout mouse embryonic fibroblasts (MEFs), indicating the essential function of PP5 in the DDR as general regulator of ATM, ATR, DNA-PK, or their substrates, as discussed below (Yong et al., 2007).

#### PP6

PP6 forms stable heterotrimers, comprising the PP6 catalytic subunit (PP6c), one of the three regulatory subunits (PP6R1, PP6R2, or PP6R3), and one of the three ankyrin repeat-containing subunits (ARS-A, ARS-B, or ARS-C; Stefansson and Brautigan, 2006; Stefansson et al., 2008). Functional analysis of PP6 showed that PP6 regulates G_1_ to S progression through controlling cyclin D1 protein expression (Stefansson and Brautigan, 2007) and mitotic spindle formation through inhibition of an essential mitotic kinase, Aurora A (Zeng et al., 2010). Knockdown of either PP6R1 or PP6c impairs DNA-PK activation, DSB repair, and IR sensitivity, indicating that PP6 has critical roles in the DDR (Mi et al., 2009b; Douglas et al., 2010; Zhong et al., 2011).

### PP2C

#### Wip1/PPM1D

PP2C belongs to the Mn^2+^/Mg^2+^-dependent PPM family. Unlike the PPP family, PP2C phosphatases are insensitive to inhibition by okadaic acid (OA) or microcystin and do not have regulatory subunits, but instead contain specific regulatory and targeting domains. Among the PP2C phosphatases, wild-type p53-inducible phosphatase 1 (Wip1), also termed PPM1D, which was originally identified in a screen for p53 target genes (Fiscella et al., 1997), has been extensively analyzed in cell cycle checkpoint contexts. Wip1 preferentially targets multiple proteins at their pSQ/pTQ motifs, which are phosphorylated by ATM, ATR, or DNA-PK. In addition, Wip1 also targets pTXpY motifs in p38 mitogen-activated protein kinase (MAPK), which activates p53 upon DNA damage, and in the uracil DNA glycosylase UNG2, which regulates base excision repair (BER; Lu et al., 2004; Takekawa et al., 2000). Wip1 has also been reported to facilitate the reversal of cell cycle checkpoint responses, returning cells to the homeostatic state after completion of DNA repair. Wip1 likely plays a role in the p53 negative feedback loop through two pathways, i.e., dephosphorylation of p38 or p53. Following DNA damage, p53 up-regulates Wip1, which then inhibits p38 via dephosphorylation at T180; inactivated p38 results in inhibition of p53 (Bulavin et al., 1999; Appella and Anderson, 2001). On the other hand, up-regulation of Wip1 reverses p53 activation by dephosphorylating p53 at S15 (Lu et al., 2005). UNG2, another target of Wip1, is phosphorylated and activated in response to UV, inducing BER activity. Wip1 also inhibits BER activity through dephosphorylation of UNG2 at T6 after completion of DNA repair (Lu et al., 2004). Importantly, mice lacking Wip1 are viable but exhibit defects in reproductive organs, immune functions, and cell cycle control (Choi et al., 2002). Knockout of Wip1 triggers p38-mediated activation of the p53, p16, and p19 pathways, leading to enhanced DDRs, promoting genomic stability, and providing resistance to transformation by oncogenes (Bulavin et al., 2004). Consistent with Wip1’s function as an oncogene, amplification of this gene has been reported in several human tumors, including breast cancer, neuroblastoma, and ovarian clear cell adenocarcinoma (Bulavin et al., 2002; Li et al., 2002; Hirasawa et al., 2003; Saito-Ohara et al., 2003). In fact, an associated checkpoint phenotype was reported; overexpressed Wip1 cells abrogated S phase and G_2_/M DNA damage checkpoints, whereas reduction of Wip1 expression enhanced the enforcement of intra-S and G_2_/M checkpoints (Lu et al., 2005).

#### PPM1G

PPM1G (also denoted PP2Cγ), a PP2C phosphatase what was originally identified as a splicing factor, has a role in the DDR. PPM1G mediates the exchange of H2A-H2B, which is implicated in the recovery from DNA damage (Kimura et al., 2006). PPM1G-knockdown cells show defects in normal cell proliferation (Allemand et al., 2007), and PPM1G-deficient DT40 cells are sensitive to DNA damage (Kimura et al., 2006), indicating the important role of PPM1G in cell growth and the DDR. Recently, another report demonstrated that PPM1G has a new function in p53 activation through the deubiquitinating enzyme USP7 (also known as HAUSP), which stabilizes the E3 ligase Mdm2 (Khoronenkova et al., 2012). In fact, following IR, ATM phosphorylates and activates PPM1G, which then dephosphorylates and inactivates USP7, leading to Mdm2 degradation and accumulation of p53.

## DDR PLAYERS ARE DEPHOSPHORYLATED BY PROTEIN PHOSPHATASES

### SENSOR KINASES, ATM, ATR, AND DNA-PK

#### PP2A

**FIGURE 1:**
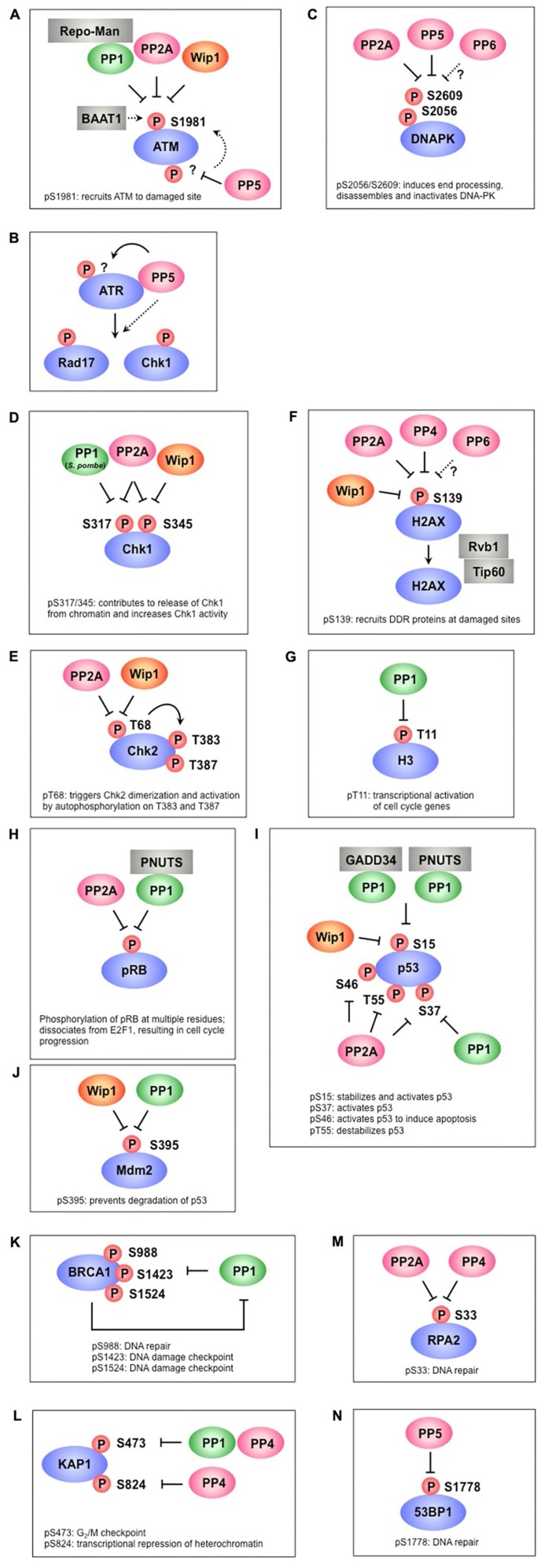
**Protein phosphatases regulate multiple phosphorylation events in the DNA damage response**. **(A)** PP1, PP2A, and Wip1 dephosphorylate ATM at S1981, which is required for the recruitment ATM to damage sites. BAAT1 protects ATM from dephosphorylation by phosphatases. PP5 is likely involved in ATM activation through dephosphorylation of ATM, which may induce ATM phosphorylation at S1981. **(B)** PP5 is required for ATR- targeted phosphorylation of Rad17 and Chk1. Although PP5 associates with ATR in a DNA damage-dependent manner, the precise mechanism remains to be determined. **(C)** PP2A, PP5, and possibly PP6 dephosphorylate multiple sites on DNA-PK, including S2056 and S2609. These sites are autophosphorylated by DNA-PK and are involved in the activation/inactivation of DNA-PK. **(D)** PP1 (*S. pombe*), PP2A, and Wip1 dephosphorylate Chk1 at S317 and/or S345, which promotes its release from chromatin and increases Chk1 kinase activity. **(E)** PP2A and Wip1 mediate the dephosphorylation of Chk2 at T68, which facilitates Chk2 dimerization and autophosphorylation at T383 and T387. **(F)** γ-H2AX (the phosphorylated form of H2AX at S139) is required for recruitment of various DDR proteins to damaged sites and is dephosphorylated by PP2A, PP4, PP6, and Wip1. Rvb1/Tip60 is implicated in the removal of γ-H2AX. **(G)** PP1 dephosphorylates H3 at T11 following DNA damage, leading to transcriptional repression of cell cycle-regulated genes. **(H)** PP2A and PP1-PNUTS dephosphorylate pRb at multiple sites, leading to inhibition of E2F1 activity and cell cycle arrest. **(I)** p53 is dephosphorylated at S15 by PP1-GADD34, PP1-PNUTS, and Wip1, resulting in p53 inactivation. S37 dephosphorylation is also mediated by PP1 and PP2A. PP2A also dephosphorylates p53 at S46 and T55. **(J)** Wip1 and PP1 dephosphorylate Mdm2 at S395, which facilitates p53 degradation. **(K)** PP1 interacts with BRCA1 and dephosphorylates multiple sites of BRCA1. In addition to acting as a PP1 substrate, BRCA1 also plays a role in PP1 inhibition. **(L)** KAP-1 is dephosphorylated at S473 by PP1 and PP4, whereas S824 is dephosphorylated by PP4. **(M)** PP2A and PP4 are required for RPA2 dephosphorylation. **(N)** Phosphorylated 53BP1 is recruited to DNA damage sites to coordinate the localization of DDR factors and promote their activation. PP5 dephosphorylates 53BP1 at S1778, leading to 53BP1 release from DNA damage sites.

PP2A has been reported to operate as a regulator of ATM (Goodarzi et al., 2004). In the absence of DNA damage, PP2A associates with and dephosphorylates ATM at S1981 (**Figure 1A**). Following DNA damage, rapid dissociation of PP2A from ATM leads to activation of ATM. Inhibition of PP2A by OA or by expressing a dominant-negative mutant of PP2Ac induces autophosphorylation of ATM at S1981 in undamaged cells without activation of ATM activity (Yan et al., 2010). Other reports demonstrated that IR induces dissociation of the B55 subunit from PP2A in an ATM-dependent manner, influencing the disruption of ATM-PP2A (Guo et al., 2002). Despite these studies, the molecular mechanism regulating the dissociation of ATM-PP2A remains to be determined.

Little is known about the role of PP2A in mediating DNA-PK. Studies have shown that the interaction of PP2A with the DNA-PK subunits Ku70 and Ku80 is induced by DSBs. Moreover, PP2A has been shown to dephosphorylate each of these proteins both *in vitro* and *in vivo* (**Figure 1C**). Further functional analyses showed that PP2A-dependent dephosphorylation of DNA-PK subunits enhances the formation of a functional DNA-PK, leading to promotion of NHEJ and DSB repair (Douglas et al., 2001; Wang et al., 2009).

#### PP1

PP1-dependent DDR regulation is partly mediated by its chromatin targeting subunit, Repo-Man, which was isolated as a PP1γ-specific interacting protein (Trinkle-Mulcahy et al., 2006; Vagnarelli et al., 2006). Studies in *Xenopus* egg extracts demonstrated that Repo-Man interacts with ATM and PP1 through distinct domains, leading to PP1-dependent regulation of ATM phosphorylation and activation (Peng et al., 2010; **Figure 1A**). Following DNA damage, the Repo-Man-PP1γ complex is released from chromatin, leading to activation of ATM at DNA damage sites.

#### Wip1

Wip1 suppresses ATM activity through dephosphorylation of ATM, resulting in restoration of ATM to its dephosphorylated state after completion of DNA repair (Shreeram et al., 2006; **Figure 1A**). Given the fact that Wip1 is constitutively associated with ATM, how Wip1 activity is regulated remains to be determined.

#### PP5

Unlike the phosphatases described above, PP5 has a role in the activation of ATM and the association between ATM and PP5 is induced by DNA damage (Ali et al., 2004). Following DSBs, ATM-mediated phosphorylation of Rad17 at pS635 and p53 at S15 is not induced in PP5-knockdown cells, which exhibit an impaired S-phase checkpoint (Ali et al., 2004). In fact, expression of a catalytically inactive PP5 mutant inhibits ATM activation, whereas wild-type PP5 does not affect the phosphorylation status of ATM at S1981 after IR exposure (Ali et al., 2004; Wechsler et al., 2004). These results suggest that PP5 is likely not involved in dephosphorylation of this site, but instead may be involved in the activation of ATM (**Figure 1A**).

Regulatory links between PP5 and ATR as well as PP5 and ATM have been demonstrated (Zhang et al., 2005). PP5 interacts with ATR in a DNA damage-dependent manner, and down-regulation of PP5 leads to defects in the phosphorylation of ATR targets, including Rad17 and Chk1, following UV or HU and an aberrant S-phase checkpoint, indicating the involvement of PP5 in ATR activation (**Figure 1B**). Whether ATR is a substrate of PP5 currently remains unknown.

PP5 interacts with and dephosphorylates DNA-PKcs (Wechsler et al., 2004; **Figure 1C**). Phosphorylation of DNA-PKcs at T2609 and S2056 is reduced in cells overexpressing PP5, suggesting that PP5 mediates dephosphorylation of DNA-PKcs.

#### PP6

DNA-PKcs associate with PP6 and all regulatory subunits, including PP6R1, PP6R2, and PP6R3, and PP6 is involved in dephosphorylation of DNA-PKcs (Mi et al., 2009b; Douglas et al., 2010; **Figure 1C**). IR enhances this interaction and promotes the import of this complex into the nucleus (Mi et al., 2009b). In contrast, other findings have indicated that the interaction between DNA-PKcs and the PP6 complex is constitutive (Douglas et al., 2010). Although it is not clear whether the interaction between DNA-PKcs and PP6 is indeed induced by DNA damage, Douglas et al. (2010) produced an attractive model in which DNA-PKcs recruit PP6 complexes to damaged sites, permitting PP6 to contribute to the dephosphorylation of H2AX, dissolution of foci, and release from the G_2_/M checkpoint.

#### BAAT1

BRCA1-associated protein required for ATM activation 1 (BAAT1), which was isolated as a BRCA1-interaction partner, is important for activation of ATM (Aglipay et al., 2006; **Figure 1A**). Expression of BAAT1 and association of BAAT1 with ATM are increased after IR. Importantly, phosphorylation of several ATM targets, including H2AX, NBS1 at S343, Chk2 at T68, and ATM at S1981, is not induced in BAAT1-knockdown cells. Defects in ATM phosphorylation at S1981 observed in BAAT1-knockdown cells could be restored by OA treatment.

### TRANSDUCER KINASES, Chk1/Chk2

#### PP2A

During the normal unperturbed cell cycle, Chk1 is phosphorylated on S317 and S345 by ATR, and in turn, phosphorylated Chk1 is antagonized by Chk1-regulated PP2A to maintain the status of Chk1 activity (**Figure 1D**). Thus, the activity of Chk1 is finely tuned in an ATR-Chk1-PP2A regulatory loop (Leung-Pineda et al., 2006).

PP2A was also reported to interact with Chk2 and regulates phosphorylation at T68 of Chk2 after DNA damage (Dozier et al., 2004; Liang et al., 2006; Freeman et al., 2010; **Figure 1E**). Studies have suggested that PP2A maintains Chk2 in an inactive state under normal conditions, while PP2A dissociates from Chk2 and permits the phosphorylation of Chk2 by ATM under DNA damage conditions. After completion of DNA repair, PP2A has a role in attenuating the DDR partly through dephosphorylation of Chk2.

#### Wip1

Wip1 binds Chk1 and dephosphorylates S345 and, to a lesser extent, S317, leading to inhibition of Chk1 activity (Lu et al., 2005; **Figure 1D**). Thus, Wip1 has a role in abrogating cell cycle checkpoints, in part through dephosphorylation of Chk1.

Wip1 also interacts with Chk2 and dephosphorylates Chk2 at T68 (Fujimoto et al., 2006; Oliva-Trastoy et al., 2007; **Figure 1E**). Knockdown of Wip1 leads to sustained phosphorylation of Chk2 at T68, promoting apoptosis in response to DNA damage. Consistent with this observation, overexpression of Wip1 antagonizes Chk2 activation. Thus, Wip1 is thought to play a negative role in DNA damage-induced apoptosis by dephosphorylation and inactivation of Chk2.

#### PP5

Upon UV irradiation, ATR-mediated phosphorylation of Chk1 at S345 is increased and maintained in PP5-depleted cells. After 24-h exposure to UV irradiation, this site is dephosphorylated to control levels, indicating that PP5 is not the only phosphatase mediating Chk1 at S345 (Amable et al., 2011). Importantly, PP5-knockout MEFs also exhibit prolonged and enhanced phosphorylation of Rad17, H2AX, and Chk1 at S317. However, contrary to this observation, one study has shown that knockdown of PP5 by antisense PP5 or ectopic expression of a catalytically inactive PP5 mutant leads to impairment of the ATR-mediated phosphorylation of Rad17 and Chk1 (Zhang et al., 2005). The precise functions of PP5 in the DDR remain to be determined.

#### PP1

The involvement of PP1 in checkpoint recovery is less well studied. However, a study in *Schizosaccharomyces pombe *demonstrated that dephosphorylation of Chk1 by the PP1 homolog Dis2 allows mitotic entry upon completion of DNA repair in G_2_ phase (den Elzen and O’Connell, 2004; **Figure 1D**). However, in human cells, knockdown of PP1 does not change the phosphorylation status of Chk1 on S317, and PP1 does not dephosphorylate Chk1 directly (Leung-Pineda et al., 2006).

### HISTONES AND HISTONE VARIANTS

#### H2AX-pS139 (γ-H2AX)

***PP4*** PP4 dephosphorylates γ-H2AX *in vitro*, and knockdown of PP4 shows persistent γ-H2AX without apparent deficiencies in DNA repair following IR, suggesting that PP4 has a direct role in the dephosphorylation of γ-H2AX (Nakada et al., 2008; **Figure 1F**). Indeed, PP4C knockdowned cells display a prolonged G2/M checkpoint arrest after IR. It is also reported that PP4 is required to repair DNA replication-mediated DNA damage and PP4 silenced cells are sensitive to DNA replication inhibitors (Chowdhury et al., 2008).

***PP2A*** In response to DNA damage, PP2A forms foci and colocalizes with γ-H2AX and dephosphorylates γ-H2AX (Chowdhury et al., 2005; **Figure 1F**). However, since repair of damaged DNA is delayed in PP2A-depleted cells, the PP2A-dependent increase in γ-H2AX may be partly due to reduced repair (Chowdhury et al., 2005; Nakada et al., 2008).

***PP6*** Down-regulation of either PP6C or PP6R1 causes extensive γ-H2AX and persistent γ-H2AX foci formation following DNA damage, suggesting that PP6 plays a role in the dephosphorylation of γ-H2AX (Douglas et al., 2010; **Figure 1F**). It is important to note that knockdown of PP6 did not affect the phosphorylation of ATM at S1981, SMC1 at S957, or Chk2 at T68 (Douglas et al., 2010). In the context of cisplatin-induced DSBs, PP6 is required for homologous recombination; thus, persistent γ-H2AX in PP6-depleted cells can be explained by delayed DSB repair (Zhong et al., 2011).

***Wip1*** A recent study reported that Wip1 binds directly to H2AX and dephosphorylates it *in vitro* and *in vivo*, leading to reverse checkpoint signaling (Cha et al., 2010; **Figure 1F**). Moreover, ectopic expression of Wip1 reduces IR-induced γ-H2AX and foci formation for several DDR factors, leading to delayed DNA repair after IR. However, whether knockdown of Wip1 affects DNA repair efficiency remains unknown.

#### Histone H3

***PP1*** We have recently identified a novel function for Chk1 as a transcriptional regulator through phosphorylation of H3 at T11 (H3-pT11; Shimada and Nakanishi, 2008; Shimada et al., 2008). This phosphorylation appears to activate the GCN5 histone acetyltransferase complex, leading to H3K9 acetylation and transcription of critical cell cycle regulatory genes, such as* cdk1 *and *cyclin B1*. Upon DNA damage, Chk1 rapidly dissociates from chromatin, H3T11 phosphorylation and H3K9 acetylation levels are reduced, and target genes are repressed. In addition to release of Chk1 from chromatin, we recently reported that activation of protein phosphatase 1 is involved in the reduction of H3-pT11 following DNA damage through suppression of T311 phosphorylation due to decreased Cdk1 activity (Shimada et al., 2010; **Figure 1G**).

### THE EFFECTOR MOLECULES pRb, p53, AND Mdm2

#### PP1

Retinoblastoma tumor suppressor protein (pRb), which is negatively regulate cell cycle progression, can interact with all PP1 isoforms (Durfee et al., 1993; Vietri et al., 2006), and PP1 dephosphorylates and activates pRb at the mitosis-to-interphase transition (Alberts et al., 1993; Durfee et al., 1993; Ludlow et al., 1993; Nelson et al., 1997; **Figure 1H**). Importantly, recent data revealed that PP1 competes with Cdks for binding to pRb (Hirschi et al., 2010). PP1 regulatory factors have also been implicated in the regulation of pRb. One of the regulatory subunits, PNUTS dissociates from PP1 under hypoxia stress, leading to activation of PP1, dephosphorylation of pRb at T821, and inhibition of cell growth (Udho et al., 2002; Krucher et al., 2006). Importantly, depletion of PNUTS in cancer cells, but not in normal cells, induces apoptosis through the activation of PP1 and its subsequent regulation of pRb (Krucher et al., 2006; De Leon et al., 2008).

p53 is phosphorylated on pS15 upon DNA damage by ATM/ATR and contributes to stabilization and activation of p53 (Dumaz and Meek, 1999; Lu et al., 2005). Phosphorylation of p53 at S37, which is also transiently up-regulated upon DNA damage, is required for p53 transcriptional activity (Dohoney et al., 2004). PP1 dephosphorylates p53 at S15 and S37 *in vitro* and *in vivo*, reducing transcriptional activity and attenuating apoptosis (Li et al., 1998, 2006; **Figure 1I**). Growth arrest and DNA damage 34 (GADD34) is known to inhibit the binding of PP1 to p53 and prevent dephosphorylation of p53 at S15 (Li et al., 1998). In addition to GADD34, PNUTS also inhibits PP1-dependent dephosphorylation of p53 at S15 and plays a role in apoptosis via regulation of p53 (Lee et al., 2007b). Thus, the association of regulators such as GADD34 and PNUTS with PP1 is required PP1-mediated regulatory activity.

p53 is also regulated indirectly through Mdm2. DNA damage-induced phosphorylation of Mdm2 at S395 by ATM attenuates the ability of Mdm2 to promote nuclear export and degrade p53 (Maya et al., 2001). Once p53 is stabilized and activated, PP1 triggers the inactivation of the signaling cascade (**Figure 1J**). Dephosphorylation of Mdm2 inhibits it autoubiquitination, resulting in stabilization, which triggers degradation of p53 (Lu et al., 2007).

#### PP2A

PP2AC physically associates with pRb, p107, and p130 *in vivo* (Cicchillitti et al., 2003; Garriga et al., 2004) and mediates oxidative stress-induced dephosphorylation of these proteins (Cicchillitti et al., 2003; Magenta et al., 2008). pRb can also be dephosphorylated by PP2A after IR, which may trigger the recruitment of pRb to replication initiation sites, thereby suppressing abnormal replication (Avni et al., 2003; **Figure 1H**).

PP2A binds to p53 following IR and dephosphorylates multiple sites, S37, S46, and T55 to control p53 activity (Dohoney et al., 2004; Li et al., 2004; Mi et al., 2009a; **Figure 1I**). Under normal cell growth conditions, p53 is phosphorylated at T55 by TATA box binding protein-associated factor 1 (TAF1), resulting in Mdm2-mediated p53 degradation (Li et al., 2004). In response to DNA damage, two reactions trigger dephosphorylation of p53 at T55 and stabilization of p53. One is mediated by the dissociation of TAF1 from p53, while the other occurs through dephosphorylation of B56γ-containing PP2A complexes (Li et al., 2007). B56γ and PP2AC levels are increased upon DNA damage, contributing to PP2A-mediated dephosphorylation of p53 at T55 (Dohoney et al., 2004; Li et al., 2007).

#### Wip1

Wip1 can dephosphorylate p53 on S15 *in vitro* (Lu et al., 2005; **Figure 1I**). In addition, ectopic expression of Wip1 decreases p53 protein levels and S15 phosphorylation, whereas knockdown of Wip1 results in increased p53 protein levels and S15 phosphorylation. Thus, Wip1 mediates dephosphorylation of p53 at S15.

Wip1 is also known to target Mdm2 at S395, promoting the stability of Mdm2 and enhancing the interaction between Mdm2 and p53 (Maya et al., 2001; Lu et al., 2007; Yamaguchi et al., 2007; **Figure 1J**).

### OTHERS

#### BRCA1

The breast cancer susceptibility gene BRCA1 plays multiple roles in the DDR, such as DNA repair and S and G_2_/M checkpoint control (Huen et al., 2010). BRCA1 has a RING finger domain and two BRCA1 terminal domains (so-called BRCT domains) involved in associations with other proteins. DNA damage induces the phosphorylation of BRCA1 at multiple residues, such as S1524 and S1423 by ATM and ATR, respectively (Cortez et al., 1999; Tibbetts et al., 2000), and S988 by Chk2 (Lee et al., 2000). BRCA1 is rapidly localized to damage sites, which contain DNA repair proteins such as Rad51. The PP1α catalytic subunit interacts with BRCA1 and dephosphorylates the sites phosphorylated by ATM, ATR, and Chk2 (Liu et al., 2002; Hsu, 2007; **Figure 1K**). Mutational research of the PP1-binding motif in BRCA1 has revealed that the interaction between BRCA1 and PP1α is important for proper relocation of BRCA1 and Rad51 to DNA damage sites and consequently is important for the DNA repair function of BRCA1. In addition, BRCA1 inhibits PP1α activity, although the precise mechanism underlying this regulatory event remains to be determined (Liu et al., 2002).

#### KAP1

Phosphorylation of KAP-1 by ATM has been implicated in chromatin relaxation at sites of DSBs (Ziv et al., 2006; Goodarzi et al., 2008), a process that is necessary to permit the recruitment of DDR factors to the damaged DNA. Lee et al. (2010) extensively studied KAP-1 as a PP4 substrate and found that PP4 controls 2IR-mediated phosphorylation sites on KAP-1, i.e., ATM-dependent phosphorylation at S824, which is important for transcriptional repression of heterochromatin, and Chk2-dependent phosphorylation at S473, which is involved in the G_2_/M DNA damage checkpoint (Lee et al., 2010; **Figure 1L**). Moreover, a recent study also revealed that PP1 mediates dephosphorylation of KAP1 at S473 and sumoylation of KAP-1 to counter the effect of ATM (Li et al., 2010; **Figure 1L**).

#### RPA2

RPA is a trimeric protein complex involved in DNA replication, DNA repair, and recombination. ATM, ATR, and DNA-PK phosphorylate one of the subunits, RPA2, and this phosphorylation event is important for the DNA repair function of the enzyme (Wang et al., 2001; Sakasai et al., 2006; Anantha et al., 2007). In addition to phosphorylation, timely dephosphorylation of RPA2 is required for the recruitment of the homologous recombination factor Rad51 and RPA2 itself to damaged sites, which facilitates DNA repair (Lee et al., 2010). PP4 dephosphorylates the RPA2 subunit at multiple sites, of which S33 seems to be critical for the function of RPA2 after DNA damage, and PP4R2 mediates the DNA damage-dependent interaction of RPA2 and PP4C, the PP4 catalytic subunit (Lee et al., 2010; **Figure 1M**). It has also been reported that PP2A is involved in the dephosphorylation of RPA2 after hydroxyurea treatment (Feng et al., 2009; **Figure 1M**).

#### 53BP1

53BP1 is phosphorylated and recruited to DNA damage sites and plays a role in the DDR, including the DNA damage checkpoint and DNA repair. PP5 has been shown to regulate the function of 53BP1 after DNA damage through dephosphorylation at S1778 and release of phospho-53BP1 foci following NCS treatment (Kang et al., 2009; **Figure 1N**).

## CONCLUSION

Spatial and temporal phosphorylation/dephosphorylation events are critical for the cellular response to DNA damage. Although much work has focused on the regulation of kinases and phosphorylation events, recent reports has revealed the involvement of protein phosphatases in the DDR and have extensively documented the physiological roles of dephosphorylation. As discussed in this review, protein phosphatases have multiple functions in the activation and inactivation of the DDR through numerous dephosphorylation events. However, several questions remain to be investigated. First, it is not clear how the activity of each phosphatase is regulated to induce dynamic dephosphorylation events following DNA damage. In the case of PP1, DNA damage triggers dissociation of PP1 and its inhibitory subunits, resulting in the activation of PP1 (Tang et al., 2008). So far, nearly 700 PP1 interacting proteins (PIPs) have been isolated; the identification of specific PIPs for each isoform may also help us to understand the individual roles of these proteins. It is possible that each associated factor is modified by phosphorylation, thereby affecting its interaction with PP1 and altering PP1 activity. It is essential to analyze the precise mechanisms of activation for each phosphatase/substrate combination. Second, in some cases, distinct phosphatases are reported to control the same substrate sites. The purpose of such multi-phosphatase regulation is unclear. Moreover, it is not known whether specific phosphatases are directly involved in the dephosphorylation of target proteins because depletion of certain phosphatase causes multiple effects, including indirect effects on the phosphorylation of target proteins.

It can be speculated that multiple phosphatases regulate different populations of targets at specific regions, such as DNA damage sites and genes whose expression is repressed or activated. Future work is required to determine the precise spatial patterns of phosphorylation events at specific time points.

Importantly, protein phosphatases may be targets for cancer therapy. Mice lacking Wip1 are resistant to spontaneous and oncogene-induced tumors (Choi et al., 2002; Hirasawa et al., 2003; Saito-Ohara et al., 2003; Bulavin et al., 2004). In addition, double knockout of ATM and Wip1 in mice rescues several phenotypes observed in ATM-null mice, such as thymic lymphomas (Darlington et al., 2012), possibly due to enhanced DDRs caused by Wip1 depletion. Although inhibition of PP1 or PP2A has some effects on reduced tumor resistance to radiation or chemotherapy (Hamilton et al., 2009; Lu et al., 2009), it is difficult to apply protein phosphatase inhibitors for cancer therapy because they also affect many other cellular events *in vivo*. In contrast, targeting specific disruption of the interaction between substrates and protein phosphatases may be useful for cancer therapy. In fact, loss of Repo-Man has been reported to reduce anchorage-independent growth of tumor cells in soft agar (Peng et al., 2010). Thus, it is important for cancer therapy to understand the mechanisms underlying the functions and regulation of phosphatases in the DDR.

## Conflict of Interest Statement

The authors declare that the research was conducted in the absence of any commercial or financial relationships that could be construed as a potential conflict of interest.
